# Molecular principles of metastasis: a hallmark of cancer revisited

**DOI:** 10.1038/s41392-020-0134-x

**Published:** 2020-03-12

**Authors:** Jawad Fares, Mohamad Y. Fares, Hussein H. Khachfe, Hamza A. Salhab, Youssef Fares

**Affiliations:** 1 Department of Neurological Surgery, Feinberg School of Medicine, Northwestern University, Chicago, IL 60611 USA; 2 High-Impact Cancer Research Program, Harvard Medical School, Boston, MA 02115 USA; 3 Faculty of Medicine, American University of Beirut, Beirut, Lebanon; 4 Neuroscience Research Center, Faculty of Medical Sciences, Lebanese University, Beirut, Lebanon

**Keywords:** Metastasis, Metastasis

## Abstract

Metastasis is the hallmark of cancer that is responsible for the greatest number of cancer-related deaths. Yet, it remains poorly understood. The continuous evolution of cancer biology research and the emergence of new paradigms in the study of metastasis have revealed some of the molecular underpinnings of this dissemination process. The invading tumor cell, on its way to the target site, interacts with other proteins and cells. Recognition of these interactions improved the understanding of some of the biological principles of the metastatic cell that govern its mobility and plasticity. Communication with the tumor microenvironment allows invading cancer cells to overcome stromal challenges, settle, and colonize. These characteristics of cancer cells are driven by genetic and epigenetic modifications within the tumor cell itself and its microenvironment. Establishing the biological mechanisms of the metastatic process is crucial in finding open therapeutic windows for successful interventions. In this review, the authors explore the recent advancements in the field of metastasis and highlight the latest insights that contribute to shaping this hallmark of cancer.

## Introduction

**Fig. 1:**
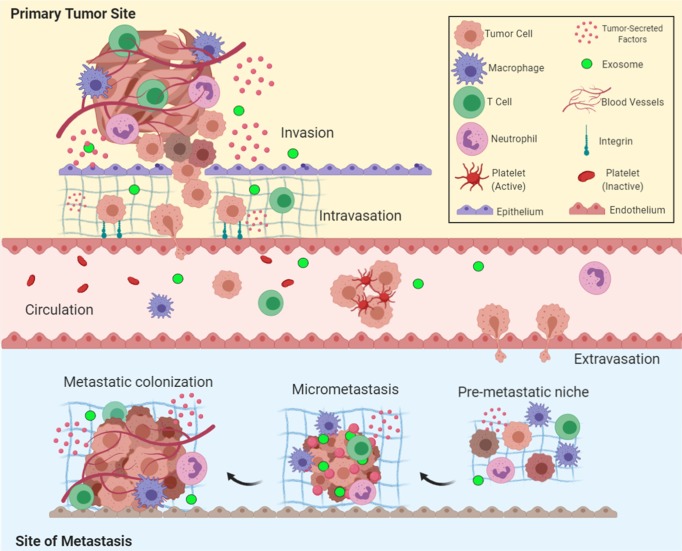
Overview of the metastatic cascade: The five key steps of metastasis include invasion, intravasation, circulation, extravasation, and colonization

The development of secondary tumors in a part of the body that is far from the original primary cancer is termed “metastasis.” Despite metastasis being the key cause of failure of cancer therapy and mortality, it remains poorly understood. In patients with cancer, large numbers of cancer cells are released in circulation daily; however, melanoma studies in animal models suggest that <0.1% of tumor cells metastasize.^1^ The development of metastases requires cancer cells to leave their primary site, circulate in the bloodstream, endure pressure in blood vessels, acclimate to new cellular surroundings in a secondary site, and escape deadly combat with immune cells.^2,3^ Hanahan and Weinberg^4^ specify that “activating invasion and metastasis” is a hallmark of cancer. Indeed, invasion of nearby tissue and seeding at distant sites to form metastases remains a central feature of cancer malignancy (Fig. 1). After all, metastasis constitutes the primary cause of death for >90% of patients with cancer.^5^ Understanding the dynamics of this process will help identify targets for molecular therapies that may halt or possibly reverse cancer growth and metastasis. Here, the authors review the recent advancements in the field of metastasis and highlight insights that contribute to shaping this hallmark of cancer.

## Dissemination and invasion

### Chromosomal instability: the initial trigger

**Fig. 2:**
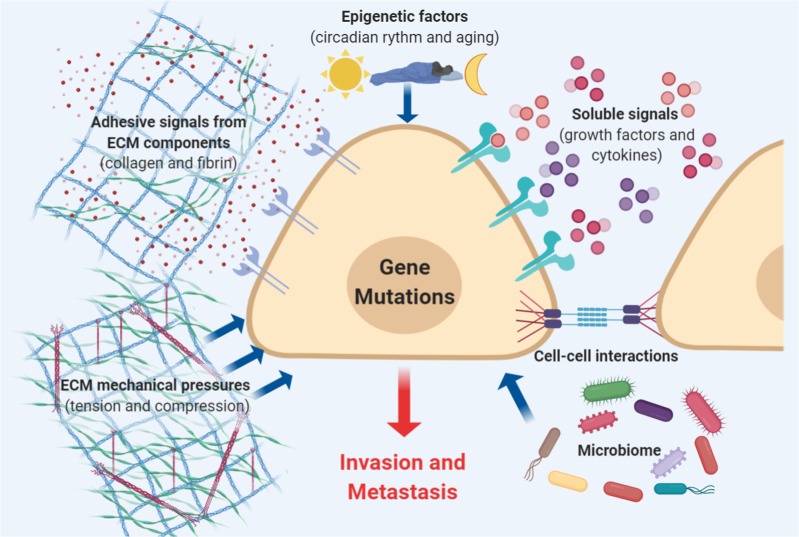
Determinants of metastasis: The activation of invasion and metastasis is triggered by epigenetic factors that are induced by environmental stimuli, such as aging and circadian disruptions; adhesive signals from extracellular matrix (ECM) components, such as collagen and fibrin; ECM mechanical pressures, including tension and compression; cell–cell interactions; soluble signals, such as growth factors and cytokines; and the intratumoral microbiota

Dissemination of cancer cells precedes the initial steps of the invasion-metastasis cascade.^6^ The cascade is the consequence of chromosomal instability that is caused by continuous errors in chromosome segregation during mitosis (Fig. 2). Faults in chromosome segregation cause the rupture of micronuclei and the secretion of genomic DNA into the cytosol, which subsequently activates cytosolic DNA-sensing pathways (cyclic GMP-AMP synthase–stimulator of interferon (IFN) genes) and downstream nuclear factor κ-light-chain-enhancer of activated B (NF-κB) signaling.^7^

Studies suggest that the nature of the primary seeding cancer cell determines the different metastatic properties with respect to growth and response to therapy.^8,9^ In vivo and in vitro studies show that metastatic cancer cells migrate individually.^10^ In humans, however, it is believed that seeding requires the joint action of a cluster of tumor cells moving together,^11^ which brings epithelial–mesenchymal transition (EMT) into the picture.

### Epithelial–mesenchymal transition: what is new?

**Fig. 3:**
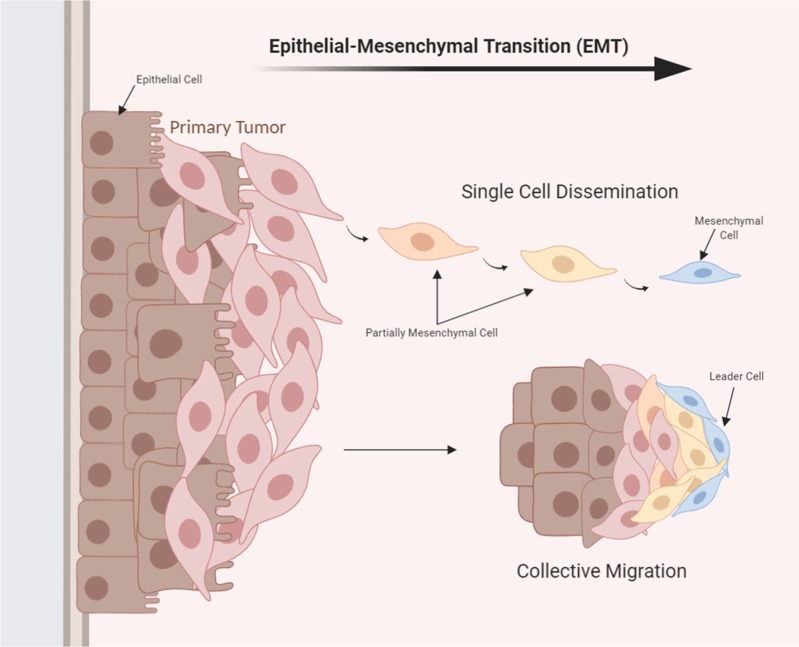
Epithelial–mesenchymal transition (EMT): EMT occurs through single-cell dissemination or through collective migration. The process consists of several transition stages between the initial epithelial cell and the invasive mesenchymal cell

EMT is the transdifferentiation process through which transformed epithelial cells develop the ability to invade, resist stress, and disseminate.^4^ Epithelial cells are immotile and tightly bound to each other and to the neighboring extracellular matrix (ECM).^12^ EMT governs the reversible biochemical alterations that permit a specific epithelial cell to attain a mesenchymal phenotype and confers epithelial–mesenchymal plasticity upon epithelial cells,^13^ which is crucial for cancer progression and metastasis (Fig. 3). However, not all cells that originate from the primary tumor site contribute to the development of metastasis. Studying the determinants of metastatic potential in a mouse model of breast cancer revealed that asparagine synthetase, a metabolic enzyme, is correlated with metastasis development.^14^ Decreasing the levels of asparagine through ʟ-asparaginase treatment or through dietary restriction decreased metastatic spread. As such, asparagine availability promoted EMT.^14^

Recently, it has become broadly understood that the EMT program is a spectrum of transitional stages between the epithelial and mesenchymal phenotypes, in contrast to a progression that involves a binary choice between full-epithelial and full-mesenchymal phenotypes.^15^ The transition of one state to another is governed by a number of growth factors^16^ and signaling pathways.^17^ Spontaneous EMT in primary tumor cells shifts between different intermediate stages with different invasive, metastatic, and differentiation characteristics.^18^ Tumor cells that express a mix of epithelial and mesenchymal phenotypes are more effective in circulation, colonization at the secondary site, and the development of metastasis.^18^ Moreover, transcriptional, chromatin, and single-cell RNA sequencing show that the various stages possess diverse cellular characteristics, chromatin landscapes, and gene expression signatures that are regulated by common and distinct transcription factors and signaling pathways. Moreover, the various EMT stages are situated in diverse microenvironments and are in contact with diverse stromal cells.^18^ For example, metastatic cells with the most pronounced mesenchymal phenotype proliferate near endothelial and inflammatory cells. These tumor cells release large quantities of chemokines and proteins to attract immune cells and stimulate angiogenesis, thus promoting the development of a unique inflammatory and highly vascularized niche.^18^ Cancer-associated fibroblasts have also been shown to drive and direct cancer cell migration through fibronectin alignment.^19^ In addition, hypoxia,^20^ metabolic stressors, and matrix stiffness^21^ trigger the EMT program in cancer cells. Transitioning is often driven by transcription factors that are programmed to repress epithelial genes and activate mesenchymal genes.^22^ Epigenetic and posttranslational modulators also play a vital role in controlling the EMT process.^15^

In recent years, there has been an important debate on whether EMT has a central role in cancer metastasis and resistance to chemotherapy.^17,23–25^ Research in lung and pancreatic cancers shows that even though EMT might not be essential for metastasis, it does contribute to chemoresistance.^23,24^ Nevertheless, more evidence is needed to completely and clearly elucidate the role of EMT in cancer progression and the metastatic process.

Although EMT might be required for metastasis initiation, the opposite process of mesenchymal–epithelial transition (MET) is needed for metastatic progression. In bone metastasis, E-selectin in the bone vasculature induces MET and WNT activation in cancer cells to drive metastatic tumor formation.^26^

### Genetic profile of metastatic cells

Metastatic cancer encompasses a diverse collection of cells that possess different genetic and phenotypic characteristics, which differentially drive progression, metastasis, and drug resistance.^27^ Hundreds of genes have been reported to determine invasive potential, suggesting that primary tumor cells exhibit a metastatic genetic signature.^28–30^ However, specific mutations can still promote invasion and metastasis in the context of some homozygous allelic expressions. Integrative clinical genomics showed that the most predominant genes that were somatically changed in metastasis included tumor protein p53 (*TP53*), cyclin-dependent kinase inhibitor 2A (*CDKN2A*), phosphatase and tensin homolog (*PTEN*), phosphatidylinositol-4,5-bisphosphate 3-kinase catalytic subunit alpha (*PIK3CA*), and retinoblastoma (*RB1*).^31,32^ Putative pathogenic germline variants were present in 12.2% of cases, of which 75% were associated with DNA repair defects.^31^

Markers that predict metastatic progression showed that advanced cancers arise from diverse cell types, which deeply affects the eventual genetic and epigenetic alterations that promote metastatic progression.^33^ Metastatic small cell lung cancer (SCLC) cells differed in the genes that they expressed.^33^ This might explain why some cancer cells respond to treatment, whereas others do not. As such, understanding intertumoral heterogeneity among different cancers can reveal the mechanisms of metastatic progression and how the cell type of origin contributes to tumor development. In colorectal cancer, cells expressing L1 cell adhesion molecule (L1CAM) confer metastasis-initiating abilities and chemoresistance. L1CAM hijacks the regenerative capacity of intestinal cells to promote metastasis.^34^ In addition, the cytotoxic immune signature and the presence of lymphatic vessels play an important role in the generation of distant metastases, regardless of genomic instability.^35^

### Metabolic profile of metastatic cells

Genetic expression that is involved in different biological processes related to metastasis is also affected by oxygen homeostasis in the tumor microenvironment.^36^ Hypoxia‐inducible factors (HIF) permit cancer cells to adapt to their cellular environment by regulating angiogenesis, EMT, invasion, metastasis, and energy metabolism.^4,37–40^ Furthermore, AXL, a receptor tyrosine kinase, has been identified as a vital mediator of HIF-dependent invasion and metastasis. In addition, HIF signaling drives the secretion of lysyl oxidase (LOX), LOX-like proteins, and exosomes, to establish a prometastatic environment within the lung and bones of patients with breast cancer.^20^ Tumor hypoxia is associated with poor prognosis in clinical scenarios;^37,41–44^ HIF‐1α and HIF‐2α expression is linked to patient mortality.^41,42^ In general, these hypoxic factors, along with others, are associated with tumor aggressiveness and resistance to therapy.^38^ Moreover, tumors with more extensive hypoxic and anoxic areas exhibit higher rates of metastasis.^45^

Metabolic differences among cancer cells lead to differences in metastatic potential. Metastatic cancer cells depend on monocarboxylate transporter 1 (MCT1) to deal with oxidative stress. MCT1 plays a major role in circulating lactate, which is a prominent energy source for metastasizing cells.^46^ As such, highly metastatic cells have increased levels of MCT1, whereas the inhibition of MCT1 decreases lactate uptake by metastatic cells and, thus, reduces their metastatic capability.^46^

Changes in ATP/ADP and ATP/AMP ratios also promote metastatic behavior. In pancreatic ductal adenocarcinoma, ECM remodeling through cellular adhesion and compression affects these ratios.^47^ Metabolomics shows that such alterations increase phosphocreatine production, which has a role in the invasive migration, chemotaxis, and liver metastasis of cancer cells.^47^

### Priming the premetastatic niche

Secondary sites do not receive invading cancer cells passively. In fact, the host microenvironment, termed the premetastatic niche (PMN), is selectively primed by the primary tumor even before the initiation of metastasis.^48^ The development of a PMN is a multistep process involving secretory factors and extracellular vesicles that induce vascular leakage, ECM remodeling, and immunosuppression.^48^ High-definition microscopes have obtained images of cancer cells sharing biological material with less malignant cells, making these cells more cancerous.^49^ Cancer cells release vesicles that carry messenger RNA transcribed from genes that are involved in cell migration and metastasis, which are then accepted by other cells.^49,50^ After host cells engulf these vesicles, human cells that did not express a malignant phenotype start to migrate faster. The transferred genes also enhance the ability of cells to invade other organs.^49^ As such, metastatic characteristics can be transferred through extracellular vesicle exchange.^49^

Primary tumors release significant amounts of exosomes that transfer invasion-promoting factors, such as microRNAs (miRNAs), to tumorigenic cancer cells.^51–53^ For example, miR-10b is carried and released by exosomes and drives metastatic properties in breast cancer cells.^54^ In addition, signaling factors mediated by exosomes activate epidermal growth factor receptor (EGFR) signaling to support cancer metastasis.^55^ Exosomes that express EGFR ligands, such as amphiregulin, tissue-type plasminogen activator, and/or annexin II, considerably increase cancer cell invasion.^56–59^ Moreover, exosomes secrete EMT inducers that stimulate EMT progression in host epithelial cells, providing them with the ability to invade and metastasize.^60–65^ Furthermore, exosomes have the ability to remodel the ECM by interacting with fibroblasts, stromal cells, and endothelial cells to degrade protease-associated components such as collagen, laminin, and fibronectin.^66^ Exosome-altered ECM exhibits increased stromal cell proliferation, cancer cell migration and survival, and tumor cell resistance to apoptotic signals. This, along with the effect of chemokines and growth factors, leads to the formation of a new microenvironment for cancer cells, immune cells, and other stromal constituents that is referred to as the PMN,^67–70^ where metastatic cells may arrest, extravasate, and ultimately colonize.^71–73^

In addition to their role in priming the PMN, exosomes exhibit properties that drive cancer cell organotropism. This metastatic bias towards certain organs stems from exosomal avidity for specific host cells.^60^ Studying the exosomal proteomic expression of bone cancer showed different integrin patterns, whereby the exosomal integrins α6β4 and α6β1 were correlated with lung metastasis, whereas exosomal integrin αvβ5 was associated with liver metastasis.^74^ Uptake of integrins in the secondary site led to the phosphorylation of Src and the expression of the proinflammatory gene *S100.*^74^ Targeting those integrins decreased exosomal uptake, and lung and liver metastasis.^74^ Other membrane proteins and lipids that are associated with ECM properties and adhesion influence the specific targeting of exosomes to their specific host cells.^74–78^ In addition, exosomal internalization by target host cells activates heterogeneous endocytic pathways such as clathrin, lipid raft, and caveolin-mediated uptake.^65,79,80^

Exosome-mediated metastasis is not solely dependent upon tumor-released exosomes. In fact, astrocyte-derived exosomes mediate the intercellular transfer of miRNAs that target the *PTEN* tumor suppressor gene to metastatic cancer cells, promoting invasion and brain metastasis.^81^ This, in turn, leads to the increased secretion of chemokine ligand 2 (CCL2), which recruits myeloid cells, enhancing the outgrowth of brain metastatic cells and reducing the effect of apoptotic signaling.^81^ Inhibition of astrocytic exosomal release prevents PTEN loss and suppresses brain metastasis.^81^

### Can metastasis be driven by epigenetic factors?

Age-related physical changes in the ECM promote or inhibit tumor cell motility, invasion, and metastasis. Alterations in the motility of immune cells lead to changes in the immune microenvironment.^82^ Elderly patients with melanoma tend to develop fewer metastases in proximal lymph nodes but have more distal metastases, with worse survival than that of younger cohorts.^83^ Through in vitro analysis, increased lymphatic permeability of endothelial membranes was shown to be the reason for this phenomenon, as lymph nodes of older patients exhibited less ECM complexity in comparison with that of lymph nodes of younger patients with metastatic melanoma.^83^ Further analysis revealed that hyaluronan and proteoglycan link protein 1 (*HAPLN1*) is responsible for controlling endothelial permeability.^82,83^ Gene knockout increased endothelial permeability and the invasive ability of disseminating melanoma cells.^83^ Other factors, such as decreased vascular endothelial-cadherin-dependent cell–cell adhesion and weak cell–ECM adhesion through α1 and β1 integrins, play a role in increasing lymph node permeability.^83^

Chromatin mutations have recently come to light as important mediators of cancer development. Chromatin alterations induce cells to gain full oncogenic characteristics.^84^ Furthermore, genetic, environmental, and metabolic conditions influence chromatin to become permissive or restrictive.^84^ Epigenetic plasticity is exhibited when permissive chromatin induces oncogenic expression to promote metastatic development.^84^

### How does the microbiome contribute to cancer metastasis?

The concept of the “tumor microbiome” originates from the fact that bacteria have been detected within tumors themselves. Although no links to patient outcomes and survival have been established, microbes have been reported to confer vulnerability to specific cancers.^85^

Bacterial translocation selectively targets tumors that have rich vascular networks and chemotactic magnetism. Anaerobic and/or facultative bacteria, specifically, vigorously survive in hypoxic tumor microenvironments.^86,87^ Tumoral bacteria are metabolically active, leading to alterations in the chemical structure of some chemotherapeutic agents and affecting the response to therapy.^88,89^ Gammaproteobacteria located in pancreatic tumors confer resistance to gemcitabine, a commonly used drug in gastrointestinal cancers.^89–91^ *Fusobacterium nucleatum* also promotes resistance in colorectal cancer by initiating autophagy and activating Toll-like receptors on cancer cells.^92^

Intratumoral bacteria further modulate the immune system. Although some bacteria stimulate antitumoral immunity, others promote immunosuppression, affecting the response to immunotherapy.^86,93–98^ The Fap2 protein of *Fusobacterium* prevents the activation of natural killer (NK) cells, protecting adenocarcinoma cell lines from NK cell antitumor activity.^99^

### Does the circadian cycle play a role in tumorigenesis?

The circadian clock controls a wide spectrum of processes in cellular physiology through metabolic and gene expression pathways.^100^ In the past decade, epidemiological studies on night-shift workers, meal timing, and exposure to light have linked alterations in circadian patterns to tumorigenesis,^101–107^ indicating that an active epigenetic mechanism may be responsible for wide-genome alterations.

Circadian clock disruptions have been correlated with cancer initiation and progression. Further alterations in transcription complexes and cellular metabolism drive cancer progression by influencing cancer cell interactions with the microenvironment.^100^ The MYC oncogene plays a role in cyclical metabolism in osteosarcoma cells, leading to increased consumption of glucose and glutamine.^108^ Moreover, a number of circadian regulating genes have been linked to MYC expression. Cryptochrome circadian regulator 2, a circadian repressor, promotes MYC degradation.^109^ Furthermore, zinc finger and BTB domain-containing protein 17 (MIZ1), a MYC-binding protein, downregulates core clock gene expression.^110^ In addition, brain and muscle ARNT-like 1 expression is inversely correlated with MYC.^110^ However, further research is needed to elucidate the mechanism through which other circadian inputs, such as nutrition, affect circadian metabolism and metastasis. CD36^+^ metastasis-initiating cells rely on palmitic acid, a dietary lipid, to promote metastasis. Blocking CD36 inhibits metastatic ability, suggesting that a high-fat diet specifically boosts the metastatic potential of metastasis-initiating cells.^111^

### Invasive cancer cells: remodeling the extracellular matrix

The ECM is a scaffold of interconnected macromolecules forming networks that encompass cells present in tissues and organs.^112^ This specialized niche alters the phenotypic properties of cells and affects their propensity to proliferate, migrate, and survive.^113,114^ Upon physiological and pathological triggers, ECM-degrading enzymes, called matrikines, are released to remodel the ECM, to re-establish an appropriate functional meshwork and maintain tissue homeostasis.^114,115^ In cancer metastasis, ECM remodeling is hijacked, leading to stromal tumorigenesis.^116–120^ A variety of major ECM components, such as proteoglycans, collagen, laminins, fibronectin, elastin, other glycoprotein, and proteinases, are involved in the invasive and metastatic processes of cancer cells.

One important step in invasion is the disassembly of the ECM and its constituents through enzymes such as matrix metalloproteinases (MMPs).^121^ MMPs play a major role in cell proliferation, survival, immune response, and angiogenesis, in addition to invasion.^122,123^ MMPs are elevated in most cancer types and are continuously associated with poor prognosis.^124,125^ Cancer cells adjust the metastatic niche to drive growth by remodeling the ECM. The changes in nutrient accessibility and metabolic reactions in tissues determine the likelihood of cancer cells to metastasize. For example, metastatic breast cancer cells metabolize pyruvate, which is plentiful in the lungs, to drive collagen-based ECM remodeling in the lung metastatic niche.^126^

Versican, a hyalectan that is present in interstitial ECM, activates EGFR signaling via its EGF-like repeats, which leads to cancer cell growth and invasion.^127,128^ Chondroitin sulfate proteoglycan 4 (CSPG4) is another ECM component that plays an integral role in stabilizing the interactions between cells in the ECM matrix. CSPG4 interacts with integrin α2β1 upon collagen type VI binding to activate the phosphatidylinositol 3-kinase (PI3K) pathway in sarcoma cells.^129^ In addition, CSPG4 forms complexes with MMP-2 and membrane type 3 MMP on the surface of melanoma cells to facilitate MMP-2 activation and eventual degradation of the ECM.^130^

Lumican is an ECM protein that organizes fibril organization and circumferential growth. It plays a major role in corneal transparency, epithelial cell migration, and tissue repair. In cancer, lumican attenuates the proliferation, migration, and invasion of breast cancer cells. It modifies cellular junctions and promotes MET^131^ through direct interactions with other ECM molecules or by the modulation of membrane receptors^132,133^ and MMP-14.^134–136^

Glypicans are proteoglycans that participate in developmental morphogenesis. They play a dual role in fostering or suppressing tumorigenesis.^114,137^ Glypican-3 exhibits a tumor suppressor phenotype. Decreased glypican-3 expression leads to the progression of malignancies, whereas its loss is associated with poor overall survival.^138^ However, elevated expression of glypican-3 correlates with reduced cancer cell differentiation and the presence of lymph node metastases in lung cancer.^139,140^ Glypican-5 overexpression also promotes tumor progression and metastasis in salivary adenoid cystic carcinoma and in rhabdosarcoma.^141–143^

Serglycin is an intracellular proteoglycan that is expressed by hematopoietic cells. Its expression drives cancer growth and metastasis.^144^ Serglycin induces EMT and chemoresistance, as well as enhances the biosynthesis of proteolytic enzymes that aid in ECM remodeling.^145^ In breast cancer, serglycin activates CD44/CREB1 signaling to enhance the secretion of transforming growth factor-β (TGF-β2) and EMT.^146^ In non-SCLC and head-and-neck cancers, serglycin activates CD44/NF-κB/claudin-1 cells and mitogen-activated protein kinase (MAPK)/β-catenin signaling to drive EMT and chemoresistance.^147,148^ Serglycin inhibition restricts the development of metastasis through decreased expression of chemokines such as CCL2.^149^ Serglycin overexpression controls the secretion of tumor-derived exosomes and their ability to trigger cancer cell invasion and metastasis.^150^

Hyaluronic acid (HA) is a glycosaminoglycan that is a principal constituent of the tumor stroma and cancer cell surfaces. It is an important EMT mediator and metastatic cancers express increased levels of HA, its CD44 receptor, and its synthase in the tumor cell microenvironment,^151^ particularly in breast, oral, prostate, and ovarian cancers.^152,153^

HA-mediated EMT enhancement is driven by the expression of zinc finger E-box-binding homeobox 1 (ZEB1) and its interaction with CD44, which in turn activates HA synthase 2 (*HAS2*) expression.^154^ *HAS2* expression regulates TGFβ-induced EMT^155^ through the expression of fibronectin, snail 1, and *ZEB1*. HAS2 has also been shown to be vital for the communication between cancer stem cells and tumor-associated macrophages (TAMs).^156^ This interaction leads to enhanced secretion of platelet-derived growth factor-BB from TAMs, which activates stromal cells and rejuvenates cancer stem cells.^156^ Inhibiting HAS2 activity via 4-methylumbelliferone limits HA synthesis and prevents metastasis in several cancer models.^156–159^

The striking effect of HA on tumor progression is highly associated with its molecular weight and interactions with other proteins in the ECM.^160,161^ Low molecular weight (LMW) HA has well‐established tumorigenic proprieties.^161,162^ In breast cancer, decreasing LMW‐HA production significantly inhibits cancer cell migration and invasion.^163^ Moreover, excess LMW‐HA in the tumor microenvironment facilitates lymphatic metastasis via disruption of intercellular adhesion among lymphatic endothelial cells.^164^ In addition, in the tumor interstitial fluid of colorectal cancer patients, LMW‐HA concentrations are increased and associated with lymphatic vessel invasion by cancer cells, and the development of lymph node metastases.^160^

Altogether, the ECM is a complex and dynamic system that is composed of a wide spectrum of cells and matrikines that participate in invasion and metastasis.

### How does autophagy contribute to cancer cell invasion?

Autophagy, the autophagosomal–autolysosomal process, is initiated by the advancement of various human cancers to metastasis. In vivo studies show that autophagy is involved in modulating tumor cell motility and invasion, cancer stem cell viability and differentiation, resistance to anoikis, EMT, metastatic cell dormancy, and escape from immune surveillance, with developing functions in forming the PMN and other metastatic facets.^165^ Autophagy inhibition does not affect cell viability, proliferation, or migration but significantly reduces cellular invasion.^166^ It was suggested that membrane-trafficking may play a critical role in the benign-to-malignant transition that is also central to the initiation of metastasis.^166^

### Can neurons initiate metastasis?

It has always been puzzling how nerves emerge in the tumor microenvironment and what their role might be. Neural progenitors from the central nervous system that express doublecortin infiltrate prostate tumors and metastases.^167^ These progenitors initiate neurogenesis, which is the process by which neurons are produced from neural stem cells.^168^ These nerve fibers in the tumor microenvironment regulate cancer initiation and dissemination, providing insights into how doublecortin-expressing neurons can be targeted for therapy.

### Does the immune environment at the primary site play a role in metastasis?

The immune microenvironment around the tumor plays a major role in dictating the metastatic potential of the disseminating cells. A study analyzed tumors from more than 800 people with colorectal cancer, comparing people whose tumors were metastatic with those who were not.^35^ The primary tumors from both groups had analogous mutation patterns in cancer genes; however, tumors that had metastasized had fewer cytotoxic T cells.^35^ In addition, the invasive ends of the spread tumor cells had reduced densities of lymphatic vessels that carry immune cells.^35^ Such changes contribute to metastasis and suggest that immunotherapies that enhance T-cell responses can stop metastasis in people with early-stage cancer.^169^ Moreover, silencing the IFN regulatory factor (Irf)-7 pathway helps metastatic cells to escape immune surveillance.^170^ In fact, a substantial number of genes that are suppressed in bone metastases are targets of Irf7 and restoration of Irf7 in tumor cells or administration of IFN led to decreased bone metastases and longer survival time.^170^ In mice that are deficient in the IFN receptor or in NK and CD8(+) T-cell responses, metastasis was faster, indicating that Irf7-driven suppression of metastasis depends on IFN signaling to host immune cells.^170,171^

### Can surgical intervention contribute to metastatic dissemination?

Sometimes, disseminated cancer cells survive and retain the ability to invade even after the removal of the primary tumor. Often, patients with pancreatic ductal adenocarcinoma develop liver metastases following surgical excision of the primary tumor.^172^ Metastasis possibly arises from dormant disseminated cancer cells that evade elimination by the immune system and are present at the time of surgery.^172^ Analyzing mouse models and tissue samples from patients with pancreatic ductal adenocarcinoma showed that dormant disseminated cancer cells do not express a cell surface molecule that elicits T-cell-mediated attacks.^172^ This phenotype is related to their inability to relieve endoplasmic reticulum stress.^172^ When this stress is lifted, disseminated cells start multiplying and invading to form metastases.^172^

Anesthetics during surgery also have an underlying mechanism in promoting metastatic dissemination. In murine models of breast cancer, sevoflurane led to significantly increased lung metastasis compared with that of propofol.^173^ Interestingly, sevoflurane increased interleukin (IL)-6 levels, which in turn led to signal transducer and activator of transcription (STAT)-3 activation and the subsequent infiltration of myeloid cells into the lung.^173^ By altering the tumor microenvironment through cytokines, anesthetics can promote cancer metastasis.

## Intravasation

Intravasation, the dissemination of cancer cells to organs through the lumen of the vasculature, is mediated actively or passively.^12,174^ This depends on the tumor type, microenvironment, and vasculature.^175^ A three-dimensional microfluidic model shows that the endothelium poses a barrier to tumor cell intravasation and is regulated by factors that are present in the tumor microenvironment.^176^ Using live-cell fluorescence microscopy and a tissue-engineered tumor-microvessel platform, a mitosis-mediated mechanism whereby tumor cells located along the vessel periphery disrupt the vessel endothelium through cell division and detach into circulation was elucidated.^177^ Furthermore, the architectural constraints of tissue impose some mechanical pressures on invading tumor cells during intravasation.^178^ Nuclear squeezing is particularly challenging on the integrity of the nucleus of the invaded cell. This causes genomic rearrangement to occur, which increases the metastatic potential.^178^

Integrins are the key cellular adhesion receptors that are involved in nearly every step of cancer progression from primary tumor development to metastasis.^179^ Altered integrin expression is frequently detected in tumors, where integrins have roles in supporting oncogenic growth factor receptor (GFR) signaling and GFR-dependent cancer cell migration and invasion.^179^ Furthermore, integrins regulate the colonization process in metastatic locations by easing anchorage-independent survival of circulating tumor cells (CTCs). Metastatic cells use E-cadherin in metastatic sites to detach, disseminate, and seed.^180^ This promotes metastatic cell survival and blocks reactive oxygen-mediated apoptosis.^180^ As such, inhibiting E-cadherin in metastatic breast cancer cells may hold therapeutic potential against breast cancer.^180^

## Circulation

### How do tumor cells survive in circulation?

The circulatory journey is harsh for most intravasating cancer cells. Interactions between CTCs and the microenvironmental components of circulation determine survival and the ability of CTCs to eventually extravasate in distant sites.^181–183^

**Fig. 4:**
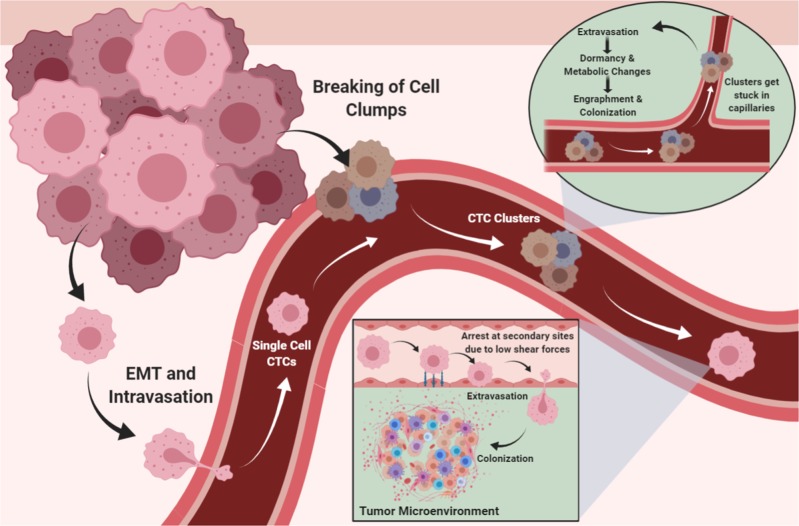
Cancer cells circulate as single units or in clusters. After arresting at secondary sites or becoming stuck in capillaries, circulating tumor cells (CTCs) extravasate and colonize their new niches. Some cells undergo dormancy as an adaptation mechanism to the new stressful environment

Most CTCs circulate as single cells, whereas others travel in clusters (Fig. 4). However, circulating clusters are much more likely to form metastases.^184^ In addition to the invading cancer cells, clusters contain stromal cells and immune components from the original microenvironment that contribute to the heterogeneity of the cluster and enhance its survival.^184–188^ Neutrophils participate in cluster formation and suppress leukocyte activation, which increases the chances of CTC survival.^189^ Moreover, the interaction of CTCs with platelets leads to the formation of a coating shield of platelets around cancer cells that prevents CTC detection by immune cells and provides the structure needed to bear the physical stresses of circulation.^190–192^

An important factor in the metastatic process is the ability of CTCs to adhere and extravasate through endothelial cells and colonize the PMN.^193^ As soon as CTCs arrest in capillaries, they either extravasate by transendothelial migration or grow within the vessel before eventual extravasation and colonization of the PMN.^194–197^

### Do circulating tumor cells interact with immune cells?

CTCs must adapt to the strict selective environment present in the lumen of the vasculature. The dissemination of CTCs is supported by close association with activated platelets and macrophages.^198^ Therefore, CTCs form heteroaggregates that sustain adhesion to the endothelium and thus contribute to metastasis.^190^ However, this belief has been challenged by showing that an increase in megakaryocytes confers some measure of protection against metastasis.^199^ In addition, neutrophils in circulation have been found to inhibit metastasis.^6,200^ Blood sampling in 70 women with advanced-stage breast cancer showed a CTC–immune cell association.^201^ The white blood cells that showed the greatest interaction were neutrophils, suggesting that neutrophil clustering with CTCs increases the metastatic potential of CTCs.^201^ The advancement of the disease in people with advanced breast cancer was faster among individuals who had CTC–neutrophil clusters when compared with that of people who lacked such clusters.^201^ Furthermore, CTCs from both CTC–neutrophil clusters and others that had not been part of a neutrophil cluster were injected into the bloodstream of tumor-free mice. A substantially increased number of metastases were found in the mice that received CTCs from CTC–neutrophil clusters. In addition, upon the eradication of neutrophils in mice with breast tumors, the number of CTC–neutrophil clusters was markedly decreased.^201^ These mice had delayed metastases in the lungs when compared with those of mice bearing breast tumors that did not have their neutrophils depleted.^201^ Moreover, the complex interchange between cancer cells and white blood cells facilitates metastasis, because metastatic cells possess sugar on their cell surface that binds to galectin-3.^202^ This enhances the ability of cells to colonize by interacting with mobilized white blood cells.^202^

### Resisting vascular forces and mechanical pressure

The journey of CTCs in the blood vessels is not easy. CTCs sense and respond to tissue mechanics and instigate brief or lasting tissue alterations, including ECM stiffening, compression and deformation, protein unfolding, proteolytic remodeling, and jamming transitions.^203^ Mechanical pressures are likely to be found during arrest of CTCs at distant sites, when exiting vessels (extravasation), and during metastatic growth. Permissive flow regimens in vascular regions, in addition to the location and efficiency of CTC lodging at distant sites, play large roles in the distant metastasis process.^204^ The passage of CTCs through the bloodstream is halted when their adhesive capacity becomes greater than the shear forces imposed on them by the blood flow.^204^ Therefore, regions with low hemodynamic flow are the regions where most CTCs stabilize and engage with endothelial cells. It is in such regions that single CTCs might sequentially form intravascular clusters.^205^ Once CTCs are fixed in the microvasculature, they are fragmented by the flow of blood. This generates immune-interacting intermediate molecules that promote extravasation and develop metastases from the surviving CTCs.^206,207^ This hypothesis was further tested in a cohort of 100 patients with brain metastases and found that these metastases formed in regions with low cerebral blood flow.^204^ Therefore, shear forces play an important role in hematogenous metastasis and in determining the location of the final arrest.

### How does the release of chemokines and cytokines help circulating tumor cells?

The migration of metastatic cells in circulation often relies on a spectrum of chemokines and complement components that direct tumor cells through the vasculature^208,209^ and metabolic factors that result in an antioxidant effect.^210^ Granulocyte macrophage colony-stimulating factor and cytokines such as IL-5, which are induced in obesity, lead to lung neutrophilia in obese mice and aid in breast cancer metastasis.^211^ In addition, when crowded, cancer cells boost the production of IL-6 and IL-8, two immune molecules that stimulate biochemical pathways and facilitate tumor migration.^212^ In mouse breast cancer models, blocking IL-6 and IL-8 receptors through experimental treatments minimized metastasis at lymph nodes, lungs, and liver compared with those of the control groups.^212^ Further data suggest that metastatic tumors induce the release of IL-1β, which induces gamma delta (γδ) T cells to release IL-17, suppressing cytotoxic CD8^+^ T lymphocytes and promoting metastasis.^213^ In addition, the loss of *TP53* in cancer cells induces the secretion of WNT ligands that stimulate the production of IL-1β, thus driving prometastatic neutrophilic inflammation.^214^

### Is tumor cell circulation contingent on the route of the bloodstream?

It is now accepted that CTCs can exploit and survive in the bloodstream during tumor metastasis.^204,207^ However, CTCs have also been found to cause distant metastases through the lymphatic circulation.^215–217^ The “sequential progression model” is the basis for excision of tumor-draining lymph nodes during surgery.^215^ Metastatic cancer cells can travel from a primary tumor to a distant site via two courses: directly through the bloodstream or through a lymph node near the primary cancer site.^218^

The biological mechanisms by which tumor cells survive and grow within lymph nodes are not yet clear. In murine models, cancer cells acclimatize to the lymph node microenvironment by shifting their metabolism to fatty acid oxidation.^218^ The signaling pathway on which the adaptation process is based is driven and activated by the yes-associated protein (YAP) transcription factor.^218^ Notably, inhibition of fatty acid oxidation or YAP signaling blocked lymph node metastasis in mice.^218^

### Diagnostics in circulation: where are we now?

Enrichment of CTCs allowed their classification and subsequent tumor analysis.^219^ CTC characterization helps reflect on the molecular foundations of metastatic tumors, whereas cell-free DNA (cfDNA) offers new genetic material for further exploration in trials.^220^ cfDNA reflects the heterogeneity of CTCs in patients with high counts of CTCs and thus enables monitoring of the metastatic burden for clinical decision-making.^221^ In addition, cfDNA profiling tracks the subclonal nature of cancer metastasis.^222^ As such, liquid biopsy of CTCs and/or cfDNA in the peripheral blood might have the potential to further the current understanding of metastasis biology.^219^ However, it is worthwhile to ponder whether currently used techniques for enrichment and detection of CTCs allow us to identify actual metastasis‐initiating cells and whether liquid biopsy can be used to investigate the effectiveness of cancer treatment.

### Targeting circulating tumor cells: can it be done?

For a long time, the low sensitivity of CTC detection assays has halted CTC elimination. In addition, the exclusion of patients with metastasis from clinical trials prevented faster progress.^223^ However, advancements in the field have changed the reigning paradigm and offered hope for future success. A photoacoustic method for direct use in patients with melanoma has been developed, allowing for the detection of very low numbers of CTCs in vivo and their subsequent destruction with laser pulses.^224^ This reflects the therapeutic potential of such approaches. In addition, distinct DNA methylation profiles are present among CTC clusters from patients and murine models with breast cancer when compared with that of single CTCs.^225^ This, along with the phenotypic differences, can be targeted in future therapeutic options.

## Extravasation

### How do circulating tumor cells extravasate?

When CTCs pass through small capillaries, they become entrapped. This either leads to microvascular rupture or forces the cell to undergo extravasation.^3^ As organs such as the liver and bone have highly permeable sinusoidal vessels, CTCs exhibit a high rate of metastasis in these organs.^12^ In other organs, extravasating cells are faced with tight barriers and basement membranes that require genetic and molecular mediation to be able to transmigrate.

Extravasation is a complex process that involves ligand–receptor interactions, chemokines, and circulating nontumor cells.^174,226,227^ Integrins, again, play a vital role in determining the sites at which extravasation and colonization occur by facilitating anchorage-independent survival of CTCs.^179^

Many have reported that cancer cell extravasation occurs in a similar fashion to leukocyte transendothelial migration.^174,228,229^ In recent years, it has been shown that cancer cells induce programmed necrosis of endothelial cells, driving metastatic cells to extravasate. Treatment with the receptor-interacting serine/threonine-protein kinase (RIPK)-1-inhibitor necrostatin-1 or endothelial-cell-specific deletion of *RIPK3* reduced endothelial necroptosis and metastatic extravasation.^230^

### Are circulating tumor cells target-specific?

**Fig. 5:**
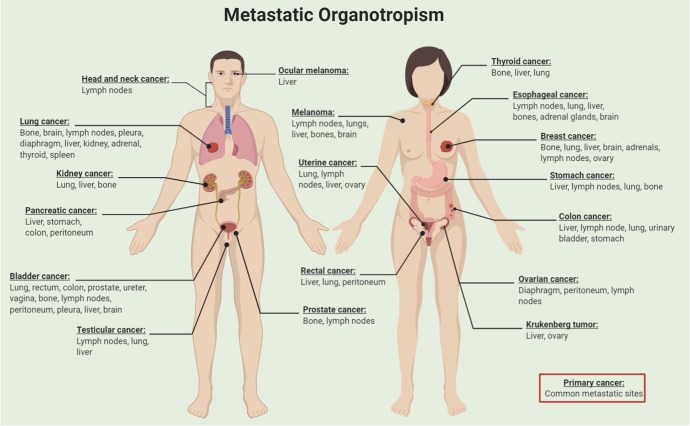
Metastastatic Organotropism: Clinical observations suggest that most cancers metastasize to specific target organs, a process known as “metastatic organotropism”

Organotropism was first touched upon by Paget as part of the “seed and soil” hypothesis.^231^ Breast cancer research has supported this hypothesis,^232,233^ with researchers elucidating the genetic basis for cancer colonization in distant organs.^234^ Moreover, the host microenvironment and the adaptive process that invading cancer cells undergo play a role in extravasation and colonization of cancer cells at specific sites.^235^ For example, breast cancer most frequently metastasizes to the bone, often after long latency, suggesting that metastatic seeds are resistant to therapy and can regrow (Fig. 5). Calcium flux, for instance, has been identified as a mechanism of crosstalk between the osteogenic niche and cancer cells, which promotes the progression of bone metastasis.^236^ Another example involves patients with postpartum breast cancer, who are at elevated risk for liver metastasis.^237^ The identification of the “weaning-induced liver involution,” which establishes a metastatic microenvironment, may account, in part, for the poor prognosis of patients with postpartum breast cancer.^237^

## Colonization

### How does the colonizing cell overcome stromal challenges?

Circulating cells that extravasate at the target site are challenged with harsh conditions that make survival difficult.^22^ A number of secreted tumor-derived factors and bone marrow-derived cells signal the formation of the PMN, in which the tumor cells colonize and grow.^12,238–240^ In addition to tumor-derived factors, exosomes play a major role (discussed earlier). Exosomes have a role in educating bone marrow progenitor cells to become metastatic.^240^ Further research showed similar results in pancreatic cancer, whereby exosomes initiated PMN formation in the liver.^73^ However, cancer cell–host cell interactions are also important for proper colonization. Hepatocytes control myeloid cell accumulation and fibrosis within the liver and thus increase the susceptibility of the liver to metastatic colonization. In murine models of pancreatic cancer, hepatocytes induce IL-6-mediated STAT3 signaling and increase secretion of serum amyloid A1 and A2 (SAA). Inhibition of IL-6-STAT3-SAA signaling prevents the establishment of a PMN and inhibits liver metastasis.^241^

Establishing a vascular network is crucial for proper metastatic colonization. Vascular mimicry drives the ability of some breast cancer cells to contribute to distant metastases through the overexpression of *SERPINE2* and *SLPI.*^242^ These two genes are overexpressed preferentially in human patients with breast cancer lung metastases, suggesting their potential for metastatic progression.^242^

Colonizing cancer cells are also capable of utilizing neuronal signaling pathways for growth and adaptation. The proximity of breast cancer cells to neuronal synapses allows cancer cells to hijack *N*-methyl-d-aspartate receptor signaling to promote brain metastasis.^243^ Protocadherin 7 is a protein that promotes the assembly of cancer cell–astrocyte gap junctions composed of connexin 43. Metastatic cancer cells use these junctions to transfer the second messenger cGAMP to astrocytes, activating the stimulator of interferon genes pathway and producing inflammatory cytokines such as IFNα and tumor necrosis factor. In turn, these factors activate STAT1 and NF-κB pathways in brain metastatic cells, thereby supporting tumor growth and chemoresistance.^244^

### Dormancy: why do cancer cells go to sleep?

By definition, cancer dormancy is an arrest phase in cancer progression that occurs during the primary tumor formation phase or after invasion into secondary sites.^245^ Metastatic dormancy specifically occurs due to the delayed acclimatization of disseminating cancer cells to their secondary niches^246^ and affects single invading cells or cancer clusters after circulation.

In many cancer survivors, dormant cancer cells are present long after radical removal of the primary tumor and are thought to be responsible for late relapses.^246^ Dormancy constitutes quiescence, angiogenic dormancy in which an equilibrium is realized between dividing and dying (vascular-lacking) cancer cells, and immune-mediated dormancy in which the tumor mass is preserved by immune cell cytotoxicity.^12,247^ Some believe that the target organ microenvironment instructs CTCs to enter dormancy, whereas others think that primary tumors pre-encode a dormancy signature on CTCs that only becomes evident when CTCs enter the host microenvironment. Another potential explanation is that early dissemination spawns CTCs that respond to dormancy-inducing signals and enter dormancy in target organs.^248^

### What are the mechanisms that govern dormancy?

Regulation of tumor cell dormancy involves reciprocal crosstalk between the environment and mechanisms that control transcriptional programs.^249^ Single-cell dormancy describes the reversible state of quiescence that the metastatic cell enters in response to stressful stimuli, while expressing the Ki67 proliferation marker.^245^ Metabolic homeostasis is maintained in the dormant state through the downregulation of two of the most well-studied pathways that are activated during oncogenesis, the RAS–MEK–ERK/MAPK and PI3K-AKT signaling cascades, which play critical roles in governing cancer cell dormancy.^250^

Factors secreted by the PMN, such as mesenchymal cell‐derived bone morphogenetic proteins (BMPs) and growth arrest‐specific 6 produced by osteoblasts, also shift cancer cells towards dormancy.^251,252^ BMP7 activates the metastatic suppressor gene N‐myc downstream‐regulated gene 1 (*NDGR1*), leading to an increase in p38 MAPK activation, cell cycle inhibitor p21 expression, and cell cycle arrest.^251^

**Fig. 6:**
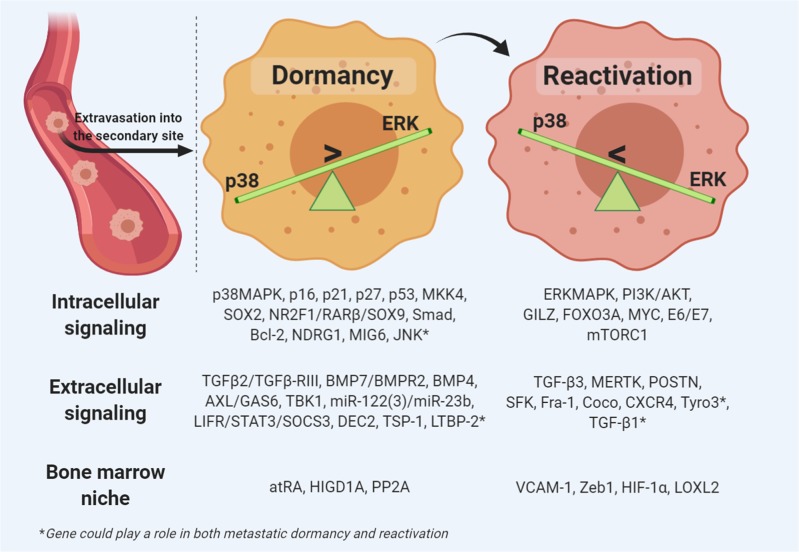
Dormancy and reactivation of cancer cells: The genetic and signaling pathways that govern cancer cell dormancy and subsequent reactivation involve intracellular signaling, extracellular signaling, and induction signals originating from the bone marrow niche

Molecular interactions between mitogen‐ and stress‐induced signaling are vital in regulating the dormancy/activation state of metastatic cancer cells. The ratio of extracellular signal‐regulated kinase (ERK1/2) to p38 MAPK regulates the cell cycle. High levels of ERK1/2 activity favor proliferation, whereas high levels of p38 favor dormancy. Increased p38 MAPK activity triggers the activation of the unfolded protein response, which upregulates activating transcription factor 6, thus promoting cell arrest and survival.^253,254^ These observations support the notion that the activation of stress signaling pathways induces a sustained state of quiescence that is linked to dormancy (Fig. 6).

### How does dormancy occur in metastatic clusters?

Dormancy in metastatic cancer clusters occurs when the rate of cellular proliferation within the cluster is equal to the rate of apoptosis. As such, the tumor cluster does not expand into micrometastasis. This balance is achieved through suppressive gene signaling, restricted angiogenesis, and/or an active immune microenvironment.^245^ Suppressive gene signaling can be achieved through the induction of differentially expressed in chondrocytes 2 (*DEC2*), a tumor suppressor gene. TGFβ induces DEC2, which inhibits cyclin-dependent kinase 4 and activates p27, forcing the cell to enter a state of quiescence.^255^ Blocking blood vessel formation through activation of thrombospondin-1^256^ or through the inhibition of chaperones, such as heat shock 27 kDa protein, pushes metastatic clusters into a dormant state.^257^ The immune system is also a major factor in cancer antiproliferation. T cells and NK cells, in addition to macrophages, clear metastatic cells through cytolysis.^258^ Dormant tumor cells express weak antigens to escape the immune system, which could be the reason behind relapse following immunotherapy.^249^

### From dormancy to activation: how does the dormant cell wake up?

Researchers have begun to understand the process that allows certain cancer cells to become dormant for periods of time and emerge later with recurring disease. These cancer cells enter a state of latency and slow division by inhibiting a WNT protein-driven signaling pathway.^246^ In addition, these cells exhibit increased levels of the stem cell genes SRY-box (SOX)-2 and SOX9, which allow for the growth of new tumors if certain conditions exist. To reduce the ability of the immune system to identify them, these dormant cancer cells downregulate the expression of immune cell-recognizable molecules.^246^ This allows tumor cells to evade an immune response until conditions allow the development of metastasis. Persistent host organ inflammation and the complementary establishment of neutrophilic extracellular traps may transform dormant cancer cells into aggressive metastases.^259^ Others believe that the shift from dormancy to activation occurs with respect to organotropism,^3^ indicating that the host microenvironment plays a role in waking the cells from their dormant state.^260^ More importantly, it has been established that high levels of ERK1/2 with respect to p38 MAPK promote reactivation.

### Cancer cell plasticity and tumor progression

Cancer cell plasticity facilitates the development of therapy resistance and malignant progression. Plasticity bestows upon cancer cells the ability to dynamically switch between a differentiated state, with limited tumorigenic potential, and an undifferentiated or cancer stem-like cell state, which is responsible for long-term tumor growth. However, researchers remain hopeful that cancer cell plasticity can be exploited therapeutically. Some have forced the transdifferentiation of EMT-derived breast cancer cells into post-mitotic and functional adipocytes by using a combination therapy of MEK inhibitors and the antidiabetic drug rosiglitazone, thereby inhibiting the metastatic process.^261^ Genome-wide in vivo screens can identify novel host regulators of metastatic colonization. In vivo studies have identified multiple genes that, when disrupted, modify the ability of tumor cells to establish metastases.^262^ Often, endovascular progenitor cells function as precursors of endothelial cells.^263^ These progenitor cells express the transcription factor SOX18 and are thus unaffected by therapies that target vascular endothelial growth factor. By ablating Notch signaling, SOX18 is inhibited, which subsequently halts melanoma metastasis in murine models.^263^

In many instances, glucocorticoids are used to treat patients with cancer-related complications. The progression of breast cancer is initiated by increasing stress hormone and glucocorticoid levels, which subsequently activates secondary site glucocorticoid receptors, enhances cancer colonization, and decreases survival rates.^202^ This suggests the use of caution when treating cancer patients with glucocorticoid therapy.

Despite the displayed effectiveness of cytotoxic chemotherapy in treating invasive breast cancer, it has been shown that the treatment displays prometastatic effects.^264^ Paclitaxel and doxorubicin trigger the production of tumor-derived extracellular vesicles in models of chemoresistant breast cancer in mice.^264^ These vesicles facilitate the colonization of tumors at metastatic sites in the lungs.^264^

### Suppressing the suppressor

**Fig. 7:**
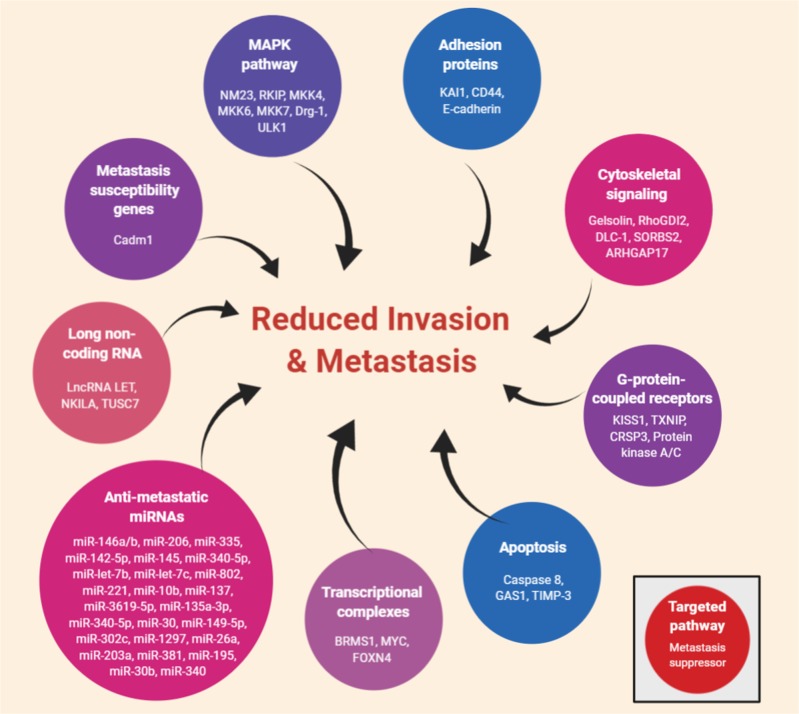
Metastasis suppressor genes that have been identified in the literature: Metastasis suppressors halt metastatic proliferation at the secondary site without changing the primary cancer. They work through oncogenic signaling pathways to suppress invasion and eventual colonization

Metastasis suppressors inhibit cancer growth and proliferation at the metastatic site without affecting the primary tumor.^265,266^ They target oncogenic pathways and proteins that are involved in invasion and eventual metastatic colonization. For example, A-kinase anchor protein 8 is a splicing regulatory factor that suppresses EMT and breast cancer metastasis.^267^ In highly metastatic cells, metastasis suppressors are usually downregulated in comparison with primary tumor cells.^265,268,269^ In the past decade, a significant number of metastasis suppressors have been identified (Fig. 7). Most notably, miRNAs that suppress oncogenes and inhibit tumorigenic signaling have been recognized and explored as potential biomarkers and targets of metastasis.^265,270–293^

## Therapeutic strategies to target the pathways of metastasis

The field of metastasis research is more than 100 years old. However, metastasis remains the primary cause of cancer-related deaths. Major obstacles lie in the lack of clinical trials that target metastasis and the lack of knowledge of the biological underpinnings that govern the metastatic process.^169,171,220,223^ Designing targeted therapies for metastatic cancer cells should take into account the genetic and phenotypic differences between parental and metastatic/circulating cells.^294^

Today, the diagnosis of metastatic cancer continues to be associated with a terminal label. Although prevention of metastasis has been demonstrated preclinically, drug development has been hindered due to poor trial design and therapeutic strategies. Advancements in immunotherapy have improved survival and patient outcomes in metastatic melanoma.^295,296^ In addition, the development of novel androgen receptor inhibitors extended the survival of metastatic prostate cancer.^297^ However, long-term follow-ups have failed to demonstrate consistency in the survival benefits of patients with metastatic breast cancer.^298^

Strategies that target pathways in the metastatic cascade have been studied and explored.^294^ The seeding of cancer cells can be targeted by inhibiting intratumoral interactions, intercellular crosstalk through ECM adhesion molecules, the release of proteases, EMT, and intravasation. However, at the time of metastasis diagnosis, cancer cells may have already seeded in the circulatory system or colonized a distant site.^220^ Therefore, targeting metastatic colonization seems to be the most plausible therapeutic strategy, as it correlates mostly with the clinical scene. Dormancy has also been studied as a potential target of metastatic colonization. Some have proposed therapies that help sustain the dormant state.^299^ Others have designed combination treatments that target G0 tumor cells. Moreover, monoclonal antibodies have been developed to target single cancer cells at this stage.^300^

In addition to CTCs (discussed earlier), the diagnostic and predictive potential of exosomes renders them key for liquid biopsies.^220,301^ In terms of targeting tumor-secreted factors, exosome affinity plasmapheresis has been developed to trap exosomes with immunosuppressive or tumorigenic material (NCT02439008); however, the trial has been terminated due to a lack of patient accrual.

The brain continues to be a special site for metastasis, as colonizing cells are offered a safe haven through the existence of the blood–brain barrier (BBB). The BBB allows the crossing of tumor cells and prevents the passage of therapeutic agents.^302^ Therefore, agents that are known to cross the BBB must be tested in brain metastasis settings and novel agents with the ability to cross the BBB must be designed.^223^

Overall, metastasis is a complex challenge that requires more than one therapeutic agent for effective inhibition. Therefore, embracing the combination therapy model and targeting multiple pathways simultaneously seems to be key to countering the significant genomic and phenotypic alterations presented by metastatic cancer cells.^303^

## Concluding remarks

Metastasis is the final frontier in cancer for which more efficacious therapies are needed. However, the development of effective treatments is contingent upon understanding the underpinnings that govern the metastatic process from start to finish. As such, exploring metastatic evolution is necessary to be able to design better therapeutics in the future.
